# Primary failure of eruption (PFE): a systematic review

**DOI:** 10.1186/s13005-018-0163-7

**Published:** 2018-03-15

**Authors:** Marcel Hanisch, Lale Hanisch, Johannes Kleinheinz, Susanne Jung

**Affiliations:** 1 Department of Cranio-Maxillofacial Surgery, Research Unit Rare Diseases with Orofacial Manifestations (RDOM), University Hospital Münster, Albert-Schweitzer-Campus 1, Gebäude W 30, D-48149 Münster, Germany; 2 Department of Orthodontics, Faculty of Health, School of Dentistry, Witten/Herdecke University, Alfred-Herrhausen-Strasse 44, 58455 Witten, Germany

**Keywords:** Eruption disorder, Orthodontics, PFE, Primary failure of eruption, PTH1R, Rare diseases, Systematic review

## Abstract

**Background:**

Primary failure of eruption (PFE) is a rare disease defined as incomplete tooth eruption despite the presence of a clear eruption pathway. Orthodontic extrusion is not feasible in this case because it results in ankylosis of teeth. To the best of our knowledge, besides the study of Ahmad et al. (Eur J Orthod 28:535-540, 2006), no study has systematically analysed the clinical features of and factors associated with PFE. Therefore, the aim of this study was to systematically evaluate the current literature (from 2006 to 2017) for new insights and developments on the aetiology, diagnosis, genetics, and treatment options of PFE.

**Methods:**

Following the PRISMA guidelines, a systematic search was performed using the PubMed/Medline database for studies reporting on PFE. The following terms were used: “primary failure of tooth eruption”, “primary failure of eruption”, “tooth eruption failure”, and “PFE”.

**Results:**

Overall, 17 articles reporting clinical data of 314 patients were identified. In all patients, the molars were affected. In 81 reported cases, both the molars and the premolars were affected by PFE. Further, 38 patients’ primary teeth were also affected. In 27 patients, no family members were affected. Additional dental anomalies were observed in 39 patients. A total of 51 different variants of the *PTH1R* gene associated with PFE were recorded.

**Conclusions:**

Infraocclusion of the posterior teeth, especially if both sides are affected, is the hallmark of PFE. If a patient is affected by PFE, all teeth distal to the most mesial tooth are also affected by PFE. Primary teeth can also be impacted; however, this may not necessarily occur. If a patient is suspected of having PFE, a genetic test for mutation in the *PTH1R* gene should be recommended prior to any orthodontic treatment to avoid ankylosis. Treatment options depend on the patient’s age and the clinical situation, and they must be evaluated individually.

## Background

Primary failure of eruption (PFE) is a rare disease with a prevalence of 0.06% [1]. PFE is defined as incomplete tooth eruption despite the presence of a clear eruption pathway. The key manifestations of PFE were first described by Proffit and Vig [2]. PFE involves partial or complete non-eruption of initially non-ankylosed teeth due to a disturbed eruption mechanism, resulting in a posterior unilateral/bilateral open bite. Orthodontic extrusion is not feasible because this procedure will cause the teeth to become ankylosed. PFE affects both primary and permanent teeth, which may erupt into initial occlusion and then cease to erupt further. Posterior teeth are most commonly affected, and typically, all teeth distal to the most mesial affected teeth exhibit the disorder [2].

PFE was further divided into three different types by Frazier-Bowers et al. [3]. In PFE Type I, the mesial to distal teeth show a similar or severe lack of eruption potential, and in Type II, the teeth distal to the most mesial affected tooth show greater but still inadequate eruption potential. Patients affected by both Type I and II PFE are diagnosed as having Type III PFE.

According to Raghoebar et al. [4, 5], localized eruption failure can be categorized into the following: (1) primary retention that is defined as an arrest of the eruption process before the crown has penetrated the oral mucosa and (2) secondary retention that involves cessation of further eruption after the tooth has penetrated the oral mucosa.

Differential diagnosis must exclude systemic or syndromic disorders such as regional cleidocranial dysplasia, regional odontodysplasia, Albers-Schönberg osteopetrosis, and GAPO syndrome. Table 1 gives an overview of rare diseases marked by tooth eruption disorders.

**Table 1:** Rare diseases featuring tooth eruption failure

Disease name	OMIM number	Orphanet number
Albers-Schönberg osteopetrosis	166600	53
Cherubism	118400	184
Cleidocranial dysplasia	119600, 216330	1452
GAPO syndrome	230740	2067
Hypodontia-dysplasia of nails syndrome	189500	2228
McCune-Albright syndrome	174800	562
Nance-Horan syndrome	302350	627
Oculodental syndrome, Rutherfurd type	180900	2709
Regional odontodysplasia		834500
Osteoglophonic dwarfism	166250	2645

Furthermore, other eruption failures such as mechanical failures of eruption (MFE) [6] or isolated ankylosis characterized by infraocclusion, immobility, metallic sound on percussion, and radiographic obliteration of the periodontal ligament space must be excluded [7].

Decker et al. [8] showed that a genetic mutation in the *PTH1R* gene is associated with PFE. The exact mechanism by which *PTH1R*-mutation leads to PFE is poorly understood [9]. Both animal and human studies have documented that PTHrP, a PTH1R ligand, is essential in the process of tooth eruption [10]. The failure of dental follicle cells to produce PTHrP causes the initially normally developed teeth to get impacted and encapsulated by a bony crypt. The activation of the cAMP/PKA pathway in tooth eruption by either ligand results in progression of tooth development and eruption. Interruption of these pathways results in ankylosis owing to upregulation of the biomineralization of cementoblasts and failure of tooth eruption [11]. Thus, clinical symptoms and confirmed mutation of the *PTH1R* gene can be used to establish a diagnosis of PFE.

To the best of our knowledge, except the study by Ahmad et al. [12], no study has systematically analysed the clinical and genetic features of PFE and its associated factors. Therefore, the aim of this study was to systematically evaluate the current literature including studies published from 2006 to 2017 for new insights and developments in aetiology, diagnosis, treatment options, and genetics to ensure early corrective diagnosis and treatment of PFE.

## Methods

A literature search of the PubMed/Medline database, including all English or German language papers published after the latest systematic review by Ahmad et al. [12] until February 2017 was performed. The reference lists of all relevant articles were also screened manually to identify further potentially relevant articles. The following search terms were used:

- “primary failure of tooth eruption”
- “primary failure of eruption”
- “tooth eruption failure”
- “PFE”

The article types included were case reports, case series, observational studies, review articles, and retrospective studies. Studies with limited data including conference abstracts and letters to journal editors were excluded.

Two calibrated reviewers (MH and LH) independently conducted the search, study inclusion, and data extraction. Any disagreement between the two reviewers was resolved by discussing with a third reviewer (SJ). According to the PRISMA guidelines [13], all records identified from the database entries were checked for duplicates. After removing the duplicates, abstracts were screened for the eligibility of inclusion. The inclusion criteria were as follows:

1. Absence of a systemic or syndromic cause
2. Clear eruption pathway (no mechanical failure, alveolar bone coronal resorbed) with apparently normal resorption
3. Involvement of the teeth distal to the most mesial affected tooth
4. No evidence of successful orthodontic extrusion of the affected tooth or teeth
5. Confirmed mutation of the *PTH1R* gene (if data were available)

**Fig. 1:**
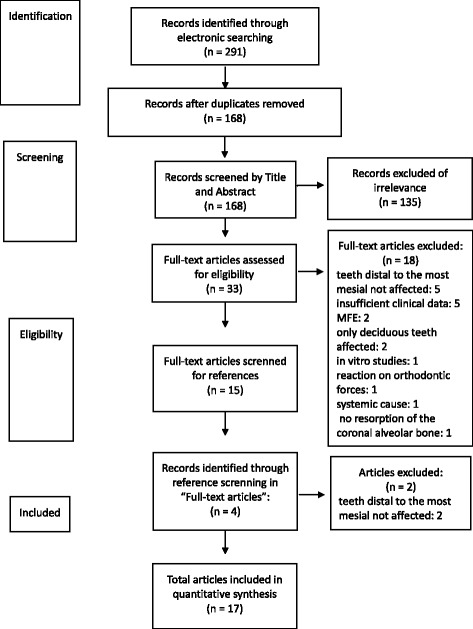
Data analyses of recorded literature for PFE according to PRISMA-Guidelines

Subsequently, full-texts were assessed for eligibility, and the references were reviewed for other reports of PFE. Using these references, full texts were assessed for eligibility. Finally, all records were analysed according to the aims of this study. The mode of literature search is summarized in Fig. 1.

## Results

### Data selection

The first literature search of the PubMed database was performed using the keywords listed in the Methods section; this search displayed 291 entries. After removing the duplicates, 168 articles remained; these were subjected to a preselection process by screening their abstracts. After the preselection, 135 articles were excluded because they were not published in English or German (*n* = 10), subjects had an associated systemic or syndromic disorder (*n* = 19), coexistence of other kinds of eruption disorders like MFE (*n* = 30), the article was an orthodontic article that did not report on PFE (*n* = 8), the article was about an animal study (*n* = 4), or the article did not report clinical or other data (*n* = 64).

Subsequently, 33 full-length articles were selected; 18 of these were excluded because of the non-involvement of the teeth distal to the most mesial affected tooth (*n* = 5), insufficient or unavailable clinical data (*n* = 5), mechanical failure of eruption (*n* = 2), only the deciduous teeth were affected (*n* = 2), contained the results of in vitro studies (*n* = 1), teeth affected by reaction to orthodontic forces (*n* = 1), existence of a systemic cause (*n* = 1), and no resorption of the coronal alveolar bone (*n* = 1).

Screening of the references from these 15 selected articles led to further inclusion of 4 articles from which 2 were rejected for non-involvement of the teeth distal to the most mesial affected tooth (*n* = 2).

### Outcome data

Finally, 17 articles reporting on PFE were included [3, 6, 8, 9, 14–26]. These articles contain case reports (*n* = 5), case series (*n* = 3), observational study (*n* = 5), and retrospective analysis (*n* = 4).

### Sex and age distribution

Overall, 314 patients were studied, with 30 female and 22 male patients. For 262 patients, the sex was not reported.

The ages of 15 female patients and 12 male patients were available; however, the age at the time of diagnosis of PFE was usually unclear. The reported ages of the female patients ranged from 8 to 58 years (mean: 24.6 years), while those of the male patients ranged from 10 to 58 years (mean: 23 years).

### Affected teeth

In all the reported cases, the molars were affected. In 118 cases, detailed clinical data were available; therefore, we could distinguish whether only molars or molars and premolars were impacted. In 81 of the 118 reported cases, both the molars and the premolars were affected by PFE (68.6%).

### Primary teeth affected by PFE

In 38 cases (24.3%), the primary teeth were affected, while in 118 patients (75.7%), the deciduous teeth were not affected. Data were unavailable for 158 cases.

### Unilateral/bilateral

Both the right and left sides were affected in 95 patients (64.1%), while only 1 side was affected in 53 cases (35.9%). No data were available for 166 patients.

### Family history

In 143 cases (84.1%), family members were also reported to have PFE. In 27 cases, none of the family members was affected by PFE (15.9%). In 144 cases, no data about PFE in family members were available.

### Types of PFE

In all, 53 patients (41.7%) were classified as PFE Type I, 40, as Type II (31.5%), and 34, as Type III (26.8%). For 187 patients, no data were available.

### Skeletal class

PFE with skeletal class I was reported in 2 cases and with class II in 5 cases, while skeletal class III was reported in 27 cases. For 280 patients, no data were available.

### Additional dental anomalies

In 39 cases, additional dental anomalies were described. These included alterations in the root morphology (*n* = 11), impacted teeth (*n* = 10), delayed eruption of further teeth (*n* = 6), hypodontia (*n* = 5), hyperdontia (*n* = 3), transposition of teeth (*n* = 2), peg-shaped teeth (*n* = 1), and MFE (*n* = 1). One author also reported alterations in the root morphology, hyperdontia, and hypercementosis in his study involving 15 patients [6]. In 70 cases, no further dental anomalies were reported, while no details regarding these data were available for 190 cases.

### Treatment

The treatment performed was reported in 22 cases, namely extraction of the affected teeth (*n* = 7), further unsuccessful orthodontic treatments (*n* = 6), alignment of the upper and lower labial segments (*n* = 1), orthodontic extrusion of the non-affected teeth (*n* = 1), overdentures (*n* = 1), and segmental osteotomy (*n* = 1). Five patients did not receive any treatment.

### PTH1R variants

In 51 cases, *PTH1R* variants associated with PFE were reported. The data are presented in Table 2.

**Table 2:** Review and data summary of PFE cases reported in the literature

Author	No. of patients	Gender, Age	First tooth affected	Family affected	Primary teeth affected	Further dental anomalies	Side affected	Typ of PFE	PTH1R Variants	Skeletal class	Treatment
Jelani et al. 2016 [14]	4	Female 12 y Female 16 y Female 19 y Female 21 y	N/A	Family affected	N/A	Hypodontia	N/A	N/A	c.611 T > A	N/A	N/A
Pilz et al. 2014 [17]	23	Female: 14 Male: 9	Posterior teeth	5 patients with affected family members	10 patients with affected primary teeth	N/A	bilateral: 20 unilateral: 3	Typ I: 4 Typ II: 6 Typ III: 13	463G > T 1016G > A 356C > T 1050-3C > G 813dupT 436C > T 1093delG 331G > T 543 + 1G > A Arg213X	N/A	N/A
Roth et al. 2014 [18]	70	N/A	N/A	N/A	N/A	N/A	N/A	N/A	c.75 + 9C > T c.310C > T c.322delT c.331G > T c.356C > T c.434A > G c.436C > A c.436C > T c.439C > T c.543 + 1G > T c.590 T > A c.636dupT c.639-2A > C c.639-2A > G c.695 T > G c.698G > A c.813dupT c.875 T > C c.1016G > A c.1036delC c.1093delG c.1142 T > G c.1148G > A c.1182C > T c.1305G > A c.1324C > G c.1355G > A c.1389 T > C c.1636G > A c.1736A > C	N/A	N/A
Frazier-Bowers et al. 2014 [9]	54	6–68 y	N/A	7 isolated cases, 47 cases family affected	N/A	N/A	N/A	Typ I in 2 families, Typ II in 8 families	c.996_997insC c.572delA	N/A	N/A
Risom et al. 2013 [20]	12	Female: 58 y Female: 29 y Female: 23 y Female: 28 y Female: 17 y Female: 57 y Female: 22 y Male: 58 y Male: 20 y Male: 27 y Male: 46 y Male: 15 y	Molar, premolar Molar, premolar Molar, premolar Molar, premolar Molar, premolar Molar, premolar Molar, premolar Molar, premolar Molar Molar Molar Molar, premolar	All cases family affected	N/A	N/A	Both Both Both Both Both Both Both Both Both Both Both Both	N/A	c.356C > T c.395C > T c.439C > T c.463G > T c.543 + 1G > A c.544-26_544-23del c.892 T > G c.947C > A c.989G > T c.1050-3C > G c.1082G > A c.1148G > A c.1348_1350del c.1354-1G > A	N/A	N/A
Rhoads et al. 2013 [19]	58	Gender: unknown Age: 24/58 available: 6–18 y	Molar affected: 20 Molar and premolar affected: 38	N/A	Primary teeth affected reported in 12 cases	Alterations in root morphology: 11 Hypodontia: 4 Delayed eruption: 6 Impacted teeth: 10 Transposition of teeth: 2	Unilateral: 27 Bilateral: 31	Typ I: 29 Typ II: 19 Typ III: 10	11 cases with genetic analysis 1092delG as new mutation identified	Class III: 18 cases	N/A
Stellzig-Eisenhauer et al. 2010 [22]	13	N/A	Molars affected: 2 Molars and premolars affected: 11	All cases family affected	One case reported	N/A	Unilateral: 5 Bilateral: 8	N/A	c.1050-3C > G c.543 + 1G > A c.436G > T	N/A	N/A
Yamaguchi et al. 2011 [21]	5	Female 36 y Female 9 y Male 17 y Male 22 y Male 19 y	Molar Molar, Premolar N/A N/A N/A	All cases family affected	One case primary teeth affected	No further anomalies	1 case both sides, 4 cases one sight affected	N/A	R383Q P119L P132L R147C	N/A	2 cases without therapy, 2 cases unsuccesful orthodontic treatment, one case segmental osteotomie
Frazier-Bowers et al. 2010 [25]	4	2 Male, 2 Female	N/A	Family affected	N/A	N/A	unilateral left: 1 unilateral right: 1 bilateral: 2	2 cases Typ I, 2 cases Typ II	c.1353-1G > A	2 cases Class III, 1 case Class I,1 case N/A	1 case: orthodontic extrusion of non affected teeth, 3 cases N/A
Decker et al 2008 [8]	13	N/A	Molars affected: 2 Molars and premolars affected: 11	Family affected	N/A	N/A	Unilateral right side: 1 Bilateral: 12	N/A	c.1050-3C > G c.543 + 1G > A c.463G > T	N/A	N/A
Sharma et al. 2016 [6]	15	Age: 6–55 years Gender: N/A	Molars affected: 5 Molars and premolars affected: 10	N/A	3 cases	hypercementosis, hyperdontia, curved root formations, delayed root development	bilateral: 7 unilateral: 8	N/A	N/A	III: 7 II: 5 N/A: 3	surgical removed (5), unsucesfull orthodontic alignment (4), no treatment (3), overdentures (1), orthodontic alignment of upper +lower labial segments (1), N/A (1)
Jain et al. 2015 [15]	1	Male 15 y	Molar and premolar affected	N/A	N/A	N/A	both	Typ III	N/A	N/A	N/A
Aruna et al. 2014 [16]	1	Male 18 y	Molar	Not affected	None	Hyperdontia 13: MFE	right	Typ II	N/A	N/A	N/A
Cohen-Lévy 2011 [23]	1	Male, 10	Molars	Family affected	Affected	No further anomalies	Left side	N/A	N/A	Class I	Extraction
Mc Cafferty et al. 2010 [24]	1	Female 8 y	Molars	Family not affected	Affected	One tooth peg-shaped	Right side	Typ II	N/A	N/A	Extraction
Proff et al. 2006 [26]	1	Gender: N/A 10y	Molars	Family affected	Primary teeth affected	One teeth with MFE	Bilateral	Typ I	N/A	N/A	N/A
Frazier-Bowers 2007 [3]	38	N/A	N/A	All cases family affected	Primary teeth affected: 8 cases	2 cases with Hyperdontia	N/A	Typ I: 17 cases Typ II: 11 cases Typ III: 10 cases	N/A	N/A	N/A

## Discussion

This study was a systematic review investigating the reported clinical data for 314 patients diagnosed with PFE. To our knowledge, after the study by Ahmad et al. [12], this is the only systematic review on PFE. Baccetti reported a prevalence ratio of 1:2.25 (male: female) [1] for PFE. Despite the considerable sample size (*n* = 314), the sex was only reported in 27 cases (15 female and 12 male patients). From these data, it was not possible to determine whether the prevalence of PFE was different in women and men.

As per Frazier-Bowers et al. [9], PFE never affects the anterior teeth owing to the autosomal dominant mutations in *PTH1R*. In 118 cases, detailed information was available; premolars as well as molars were affected in 81 cases. No studies reported PFE in teeth other than molars and premolars, indicating that PFE only affects these teeth.

Deciduous teeth were impacted by PFE in only 38 patients, and 118 patients reported that their primary teeth were not affected by PFE. Hence, it can be said that PFE affects both dentitions.

In the study by Ahmad et al. [12], 13% of the patients had hypodontia; this percentage was substantially higher than that in the normal population. In our review, out of 314 patients, only 5 were affected by hypodontia. The dental anomaly most commonly reported in our study was alteration in the root morphology (*n* = 11). The small number of reported cases indicates that additional dental anomalies like hypodontia are not significantly associated with PFE.

Since Decker et al. [8] identified a mutation in the *PTH1R* gene, 51 mutations of the *PTH1R* gene responsible for PFE were found in a review of the current literature. In addition to PFE, *PTH1R* mutation is also associated with four more clinically overlapping human disorders per the type of mutation: Jansen’s metaphyseal chondrodysplasia, Eiken syndrome, which is a skeletal disorder, Blomstrand osteochondrodysplasia, and Ollier disease [14]. Nevertheless, it is unclear whether only mutations in the *PTH1R* gene cause PFE because not all patients with PFE had the *PTH1R* mutation [18]. It has been reported that viral attacks on the nerve paths or mumps may lead to the development of dental disorders as well as eruption [27, 28], but there is a lack of evidence in this regard.

Based on their study conducted in 2006, Ahmad et al. [12] conclude that a strong family history of PFE is a risk factor for developing PFFE, while Rhoads et al. reported that the previously reported prevalence rates of 10% to 40% for familial PFE cases are expected to increase as more information about the genetic makeup of patients diagnosed with PFE is obtained [19]. In our systematic review, 143 patients were reported to have a family history of PFE, while 27 patients had no family history of PFE. In 144 cases, no further information about the family members was available. Considering the 170 patients who gave further detailed information about the occurrence of PFE among their family members, almost 85% had a family member affected by PFE. Absence of PFE in the family history may be explained by spontaneous mutations [3].

Sometimes, it is difficult to distinguish PFE from other eruption disorders like ankylosis. Based on the results of this study, the ratio of bilateral or unilateral side being affected by PFE is 1.8:1. This could help differentiate PFE from isolated ankylosis, which affects usually only one arch [19]. However, further significant data are needed to confirm these results.

An infraoccluded supracrestal first molar seems to be the hallmark of PFE [19], and all teeth distal to the most mesial tooth are affected by infraocclusion and PFE [2]. Nevertheless, in some patients, it is unclear whether PFE is present or not. If a patient is suspected of having PFE and other eruption failures like MFE, isolated ankylosis or systemic/syndromic disorders must be excluded, and a genetic test for mutations in the *PTH1R* gene must be recommended to prevent incorrect treatment [25]; especially, orthodontic extrusion must be avoided as it can lead to ankylosis.

Treatment provision was only reported in 22 cases. As per the study by Proffit and Frazier [3] the practice of extracting the teeth affected by PFE is correct. Generally, treatment depends on the patient’s age and the clinical situation [23]. In young patients, direct or indirect composite build-ups could ensure occlusal stability and preserve alveolar bone level until an implant placement is possible [29]. In adult patients with only mild infraocclusion, no treatment is required; however, regular observation is necessary [23]. In addition, prosthetic build-ups with a maximum height of 5 mm can be used to minimize the lateral infraocclusion [30].

In addition to the extraction of teeth affected by PFE, further surgical measures such as segmental osteotomy to surgically reposition the teeth into occlusion [3] and distraction osteogenesis to correct the extreme posterior open bite may also be performed [31], however, few successful cases have been reported. Often, a removable prosthesis is the only feasible therapeutic option [32]. All in all, only a few cases describing treatment options were reported. Therefore, treatment options should be evaluated by clinical studies in future.

## Conclusions

Infraocclusion of the posterior teeth, especially if both sides are affected, seems to be the hallmark of PFE. If a patient is affected by PFE, all teeth distal to the most mesial tooth are also affected by PFE. Primary teeth can also be impacted; however, this may not necessarily occur. If PFE is suspected in a patient, a genetic test for mutation in the *PTH1R* gene should be recommended prior to any orthodontic treatment to avoid ankylosis. Treatment options must consider the patient’s age and the clinical situation, and they must be evaluated individually.

## Abbreviations

MFE: Mechanical failure of eruption
PFE: Primary failure of eruption
